# Mediated effect of cognitive behavioural therapy on depression outcomes

**DOI:** 10.1186/1745-6215-14-S1-P112

**Published:** 2013-11-29

**Authors:** Lang'o Odondi, Glyn Lewis, Willem Kuyken, Chris Metcalfe, Nicola Wiles

**Affiliations:** 1 University of Bristol, Bristol, UK; 2 University of Exeter, Exeter, UK

## Introduction

Understanding how the intervention works is a primary objective in most pragmatic trials. Although good evidence exists for the effectiveness of cognitive behaviour therapy (CBT), there is little evidence presently that cognitive factors, such as dysfunctional attitudes (DAS) or meta-cognitive awareness (MAQ), mediate the effect of CBT on depression outcomes. Standard regression methods adjusting for such mediators produces biased estimates. Using data from the CoBalT trial, we use a ‘causal inference' approach to examine the mechanism through which CBT affects depression outcomes.

## Methods

The proportion of the causal effect of CBT on depressive symptoms mediated by DAS and MAQ was estimated (with 95% confidence intervals) using the ‘potential outcomes' approach. The outcome was the Beck Depression Inventory (BDI-II) score at 12 months. Mediators were measured at 6 months post-randomisation.

## Results

Approximately 25% of the treatment effect observed at 12 months was mediated through changes in DAS (24%; 95%CI: 16, 41) or MAQ at 6 months (28%; 95%CI: 20, 52). This equated, on average, to a one point reduction in the difference in mean BDI-II scores between treatment groups. Sensitivity analyses suggested that a modest degree of ‘hidden' confounding (ρ≅0.3) would violate the key assumption of no confounding between any of the three sets of variables.

## Conclusion

Changes in cognitive variables contributed a modest proportion of the observed treatment effect. Causality is intuitively counterfactual and use of potential outcomes provides valid causal inference. However, sensitivity analyses suggested that our findings need to be interpreted with caution as hidden confounding may explain the mediated effect.

